# Northern blotting of endogenous full-length human-specific LINE-1 RNA

**DOI:** 10.1093/biomethods/bpae036

**Published:** 2024-05-28

**Authors:** Maisa I Alkailani

**Affiliations:** College of Health and Life Sciences, Hamad Bin Khalifa University, Qatar Foundation, Doha, P.O. Box 34110, Qatar

**Keywords:** L1-Hs, retrotransposon, northern blot, radiolabeling, RNA probes

## Abstract

LINE-1 belongs to a family of DNA elements that move to new locations in the genome in a process called “retrotransposition.” This is achieved by a copy-and-paste mechanism with the aid of an RNA intermediate. The full-length LINE-1 is responsible for most retrotransposition activity in the human genome. Detecting the active LINE-1 RNA at the endogenous level is challenging due to its small percentage among inactive copies and its different forms of transcripts. Here, we describe a method of designing RNA probes to detect active LINE-1 by northern blotting and use optimized conditions and tools to make the detection practical. This method uses a classical long RNA probe and provides an alternative way to detect LINE-1 RNA using multiple short RNA probes.

## Graphical Abstract

**Graphical Abstract:**
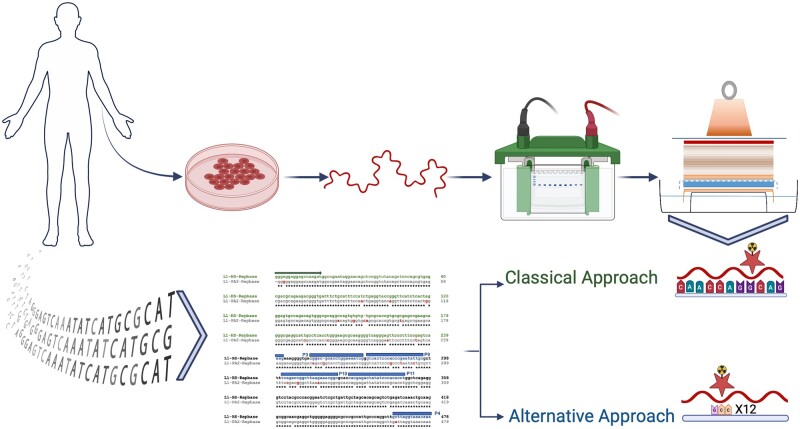

## Introduction

LINE-1 (L1) is an autonomous, protein-coding retrotransposon that moves from one genomic location to another by a copy-and-paste mechanism [1]. Due to point mutations, rearrangements, or truncations, of the 500,000 copies of L1 in the human genome, only 0.02% represent active elements, termed “competent” human-specific L1 (L1-Hs) elements [2, 3]. In the L1 subfamilies classification, L1-PA refers to insertions in primates with older subfamilies designated with higher numbers (e.g. L1PA17- L1PA2) [4]. L1-Hs belongs to the youngest subfamily and refers to insertions occurring in humans and is equivalent to L1PA1 [5]. Insertions amplified over the evolutionary time either got lost or became fixed in the population with higher rates of sequence divergences in older subfamilies than the younger ones [6]. Each full-length L1-Hs element is 6 kb long and is transcribed by its intrinsic sense promoter [1]. This promoter is in the L1 5′-untranslated region (UTR), occupying the first 100 of its 900 bp [7]. The mRNA transcribed by this promoter is mostly capped and can retrotranspose after successfully translating L1 proteins (ORF1p and ORF2p) [8]. The L1 sequence is relatively conserved throughout L1 evolutionary history except for the 5′-UTR, which differs between L1 lineages of the same species and between humans and different species [4].

Due to the small percentage of competent L1 elements in the genome, their detection by standard methods remains challenging. Northern blotting is the standard method to detect L1 mRNA either by its exogenous expression in cells or endogenously in specimens [9]. Among other techniques used to study the expression of one or a few genes, such as ribonuclease protection assay and reverse transcription-quantitative polymerase chain reaction (RT-qPCR), northern blotting is advantageous for looking into L1 transcripts. Some advantages include determining RNA expression level and the transcript size, detecting RNA degradation, splice, or processing products, and isolating different transcripts from heterogeneous pools of RNA [10]. The detection of the competent L1 elements endogenously in somatic cells is complicated by the presence of 5′- or 3′-truncated transcripts, chimeric transcripts, and spliced L1 transcripts [11]. Therefore, in their previous attempts to detect L1 RNA (either exogenously by expressing L1 in cells or endogenously in cells and tissue samples), researchers had to select Poly A-expressing elements to reduce the background noise of other populations [9, 11–14]. Here, we describe a method to detect endogenous full-length L1 using different RNA probes. This method was described briefly in the author’s paper Alkailani *et.al*. [15] to demonstrate BRCA1 (breast cancer type 1 susceptibility protein) transcriptional regulation of L1. The article provided evidence from different cell models of BRCA1-promoted accumulation of retrotransposon RNA by inducing the transcription of active families of retrotransposons (including L1-Hs) and their insertion into the genome [15]. The human specificity of this activity was confirmed using PCR primers designed to target the sequence of L1-Hs at sites of mismatches with L1-PA different subfamilies [15]. Northern probes were designed using the L1-Hs consensus sequence to detect the L1 5′-UTR (mostly un-conserved) region to validate the observed findings and to exclude the possibility of relating the effect of BRCA1 on L1 activity to an aggregation of multiple L1-PA RNAs [15].

## Experimental strategy

Two approaches were employed to detect full-length L1: a classical approach in which long probes are designed to detect the 5′ first 486 bp and a new alternative approach equipped with multiple short probes designed to cover the 900 bp of L1 5′-UTR. In our methods, the classical protocol was adapted from the previously described protocol with a different probe sequence [11], and the new protocol was designed to provide an alternative method for L1 detection. Some technical steps were adapted to the methods for optimization needs and to make the detection more practical (indicated as they appear in the methods).

## Materials and methods

### Reagents

ES2 cells (RRID: CVCL_AX39).

HeLa cells (ATCC Cat# CCL-2, RRID: CVCL_0030).

Lipofectamine™ RNAiMAX reagent (Invitrogen, 13778075).

TRIzol (Invitrogen, 15596018).

DNase I (Qiagen, 79254).

[γ-^32^P]ATP (10 mCi/ml; Perkin Elmer, BLU002A500UC).

[α-^32^P]dCTP (10 mCi/ml, 3000 Ci/nmol; Perkin Elmer)

dATP, dGTP and dCTP (Applied Biosystems).

T4 polynucleotide kinase (NEB, M0201S).

Random hexamer deoxynucleotide primers (IDT DNA).

20X SSC Buffer (Sigma).

Taq DNA polymerase (BioBasic).

Qiagen PCR cleanup kit (Qiagen).

Kelnow DNA polymerase (NEB).

RiboRuler high-range RNA ladder (ThermoFisher).

Formaldehyde.

DEPC-treated water.

### Buffers

3× loading dye: 50% formamide, 33.3% formaldehyde, 1.7× MAE, 13 μg EtBr, and 0.02 (w/v) % bromophenol blue (to be made fresh every use).

10**×** MAE buffer (pH 7.0): 200 mM 3-(N-morpholino)propanesulfonic acid (MOPS), 50 mM NaAce, 10 mM Ethylenediaminetetraacetic acid disodium salt dihydrate (EDTA). The volume was adjusted to 1 l by adding RNase-free H_2_O, pH was NaOH adjusted to 7, and the solution was filtered and stored wrapped in aluminum foil at room temperature.

Hybridization buffer: 30% formamide, 1× Denhardt’s solution, 1% SDS, 1 mM NaCl, 1 μg/ml salmon sperm DNA, RNase-free water.

5**×** random priming buffer: 250 mM Tris–Cl pH8.0, 25 mM MgCl2, 100 mM NaCl, 10 mM DTT, 1M HEPES pH 6.6.

Stop buffer: 50 mM Tris–Cl pH 7.5, 50 mM NaCl, 5 mM EDTA, 0.5% w/v SDS.

### Tools and equipment

10 cm^2^ cell culture plates.

Eppendorf tubes.

Nanodrop system (ThermoFisher).

Milli-Q^®^ Type 1 ultrapure water system.

Microwave.

Positively charged nylon membranes (Roche, 11417240001).

Stratalinker 2400.

Sanitary (or equivalent) absorbent pads (commercially available).

Blot absorbent filter paper sheets.

Saran wrap or Parafilm.

Gel Doc imaging system (BioRad).

Hybridization oven.

Capped glass cylinders for hybridization.

G25 Sephadex column (GE Life Sciences).

A Typhoon Trio phosphorimager.

X-ray films with an intensifying screen.

Electric kettle.

### Probes design and preparation

The design and preparation of the northern blot probes below were described briefly in the author’s paper [15].

#### Long probes used in the classical approach

**Figure 1:**
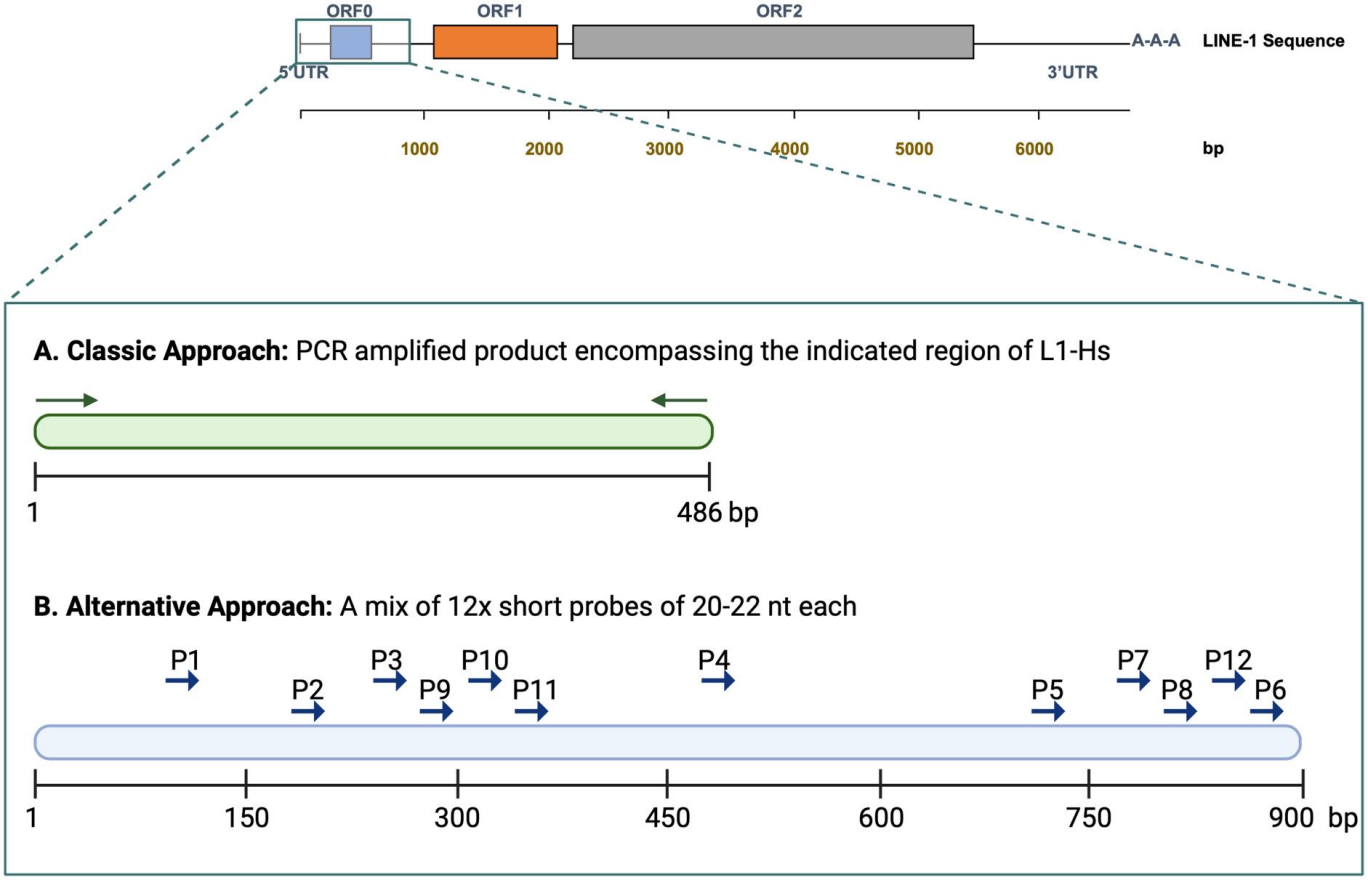
A schematic illustration to show the RNA probe design strategy based on the L1-Hs sequence. Note: The exact nucleotide sequences are included in Table 1 and displayed in the Supplementary Appendix. P1–12 denote Probes 1–12.

Based on the Repbase L1-Hs and GAPDH DNA sequences, probe DNAs were Taq DNA polymerase (BioBasic) PCR amplified from the HEK293T cellular DNA. The primer pairs used in the amplification are included in Table 1. The primers were designed to have L1-Hs product encompassing the 5′ most 486 bp of the resulted transcript as displayed in Fig. 1 and section 1 of the Supplementary Appendix.

**Table 1.** Sequences of the northern blot short probes and primers used to generate the long probes.

Short probes	5′–3′ sequences	Reverse complementary sequences
L1-HS-5UTR-P1	CTAGTGAGATGAACCCGGTACC	GGTACCGGGTTCATCTCACTAG
L1-HS-5UTR-P2	CTTTGACTCGGAAAGGGAACTC	GAGTTCCCTTTCCGAGTCAAAG
L1-HS-5UTR-P3	CGATTTTCCAGGTGCGTCCG	CGGACGCACCTGGAAAATCG
L1-HS-5UTR-P4	CGGCTGCTTTGTTTACCTAAGC	GCTTAGGTAAACAAAGCAGCCG
L1-HS-5UTR-P5	GGTCAGGGGTCAGGGACCCA	TGGGTCCCTGACCCCTGACC
L1-HS-5UTR-P6	TGATGTACAGATGGGTTTTCGG	CCGAAAACCCATCTGTACATCA
L1-HS-5UTR-P7	GGTGCCTCCCAGTTAGGCTG	CAGCCTAACTGGGAGGCACC
L1-HS-5UTR-P8	TGTCAGTGTGCCCCTGCTGG	CCAGCAGGGGCACACTGACA
L1-HS-5UTR-P9	GCAATATTCGGGTGGGAGTGAC	GTCACTCCCACCCGAATATTGC
L1-HS-5UTR-P10	GCCGTTTCTTAAGCCGGTCTGA	TCAGACCGGCTTAAGAAACGGC
L1-HS-5UTR-P11	AGGTGTGGGATATAGTCTCGTG	CACGAGACTATATCCCACACCT
L1-HS-5UTR-P12	TTGGAATACCCTGCCGTGTGAG	CTCACACGGCAGGGTATTCCAA
GAPDH-P1	GAGAGAACAGTGAGCGCCTAGT	GCGCTCACTGTTCTCTC
GAPDH-P2	ACCCGTTGACTCCGACCTTC	GAAGGTCGGAGTCAACGGGT
GAPDH-P3	TACGACTGCAAAGACCCGGAGC	GCTCCGGGTCTTTGCAGTCGTA
GAPDH-P4	GGACCTCCATAAACCCACTTCT	AGAAGTGGGTTTATGGAGGTCC
GAPDH-P5	GCTACAGAAAGGTCAGCAGCTA	TAGCTGCTGACCTTTCTGTAGC
GAPDH-P6	GGCTTTGTGAGGAACTGGGAGA	TCTCCCAGTTCCTCACAAAGCC
GAPDH-P7	ACGAAGCCCTTCCAGGAGAAG	CTTCTCCTGGAAGGGCTTCGT
GAPDH-P8	CTCAGCACAAAGAACCCTCAGG	CCTGAGGGTTCTTTGTGCTGAG
GAPDH-P9	CCAGGGCCGGCAGATGTCTTA	TAAGACATCTGCCGGCCCTGG
GAPDH-P10	GGACCTCCTGTTTCTGGGGACT	AGTCCCCAGAAACAGGAGGTCC
GAPDH-P11	GAAAGAAAGCGTCCCCCACCTA	TAGGTGGGGGACGCTTTCTTTC
GAPDH-P12	CAAGGCTGTTTGGAGGCCCTAC	GTAGGGCCTCCAAACAGCCTTG

**Long probe primers**	**5′–3′ Sequences**	**Reverse complementary sequences**

L1-HS-Forward	GGGAGGAGGAGCCAAGAT	
L1-HS-Reverse	CCGGCTGCTTTGTTTACCTA	TAGGTAAACAAAGCAGCCGG
GAPDH-Forward	ACCACAGTCCATGCCATCAC	
GAPDH-Reverse	GCTTGACAAAGTGGTCGTTG	ACGACCACTTTGTCAAGC

#### Short probes used in the alternative approach

Available L1-Hs and L1-PA full sequences were extracted from Repbase [16] and DFAM [17] databases. Clustal Omega alignments were used to design 12 different L1-Hs short probes of 20–22 nt each. These probes were selected from the first 900 nt in the 5′-UTR of L1-Hs RNA to bind different regions precisely where mismatches with L1-PA sequences were detected. Figure 1 and the Supplementary Appendix display the locations of the designed probes in the consensus L1-Hs sequence using their reverse complementary sequences. The exact mismatches with L1PA subfamilies are included in section 2 of the Supplementary Appendix for the L1PA2 sequence and section 3 for the others.

Twelve GAPDH probes were designed across its DNA sequence and prepared similarly to be used as internal controls. Table 1 contains the list of probe sequences used in the following protocol.

### RNA harvest

1. Cells were seeded in 10 cm^2^ dishes, and the next day, they were transfected with control siRNAs or siRNAs against BRCA1 using Lipofectamine™ RNAiMAX reagent. The cells were kept in culture 72 h after transfection until 80%–90% confluent (HeLa cell line was used for technique optimization purposes, and ES2 cell line was used for the experiment to demonstrate the effect of BRCA1 on L1 in ovarian cancer model).
2. The dishes were PBS-washed (at room temperature), and TRIzol reagent was added directly to them (1 ml per plate). Cell lysates were collected by pipetting up and down several times and placed in Eppendorf tubes.
3. RNA was isolated and treated with DNase I following the manufacturer’s directions and stored in a freezer at −80°C until used.

### RNA sample fractionation and gel electrophoresis

1. Gels were prepared each by adding 1 g of agarose to 85 ml Milli-Q water, dissolved by microwave heating, and cooled down to about 45°C.
2. 10 ml of 10× MAE buffer and 5 ml of formaldehyde were added to the prepared gel.
3. Gel was poured into a 10 × 15 cm slab gel box with a well comb to generate about 6-mm wells, and the gel was allowed to solidify.
4. RNA samples were taken from the −80°C freezer and were thawed on ice.
5. The RNA concentration was measured using the Nanodrop system, and each test sample needed about 50–100 μg RNA.
6. Under the chemical fume hood,
  - the 3× loading buffer and the appropriate amount of nuclease-free water were added to each sample to have equal volumes across samples (as the RNA concentration differs). Note: Make sure to keep the volume within the well capacity.
  - The RNA ladder was prepared by mixing 6 µl of RiboRuler high range with 3 µl of 3× loading buffer.
7. Samples and ladder were heated at 70°C for 10 min and were placed immediately on ice for at least 5 min.
8. Samples were loaded on the gel and run in 1× MAE at 110 Volt (for about 2 h) until the dye reached the bottom of the gel.

Note: Due to issues related to the RNA quality or the casted gels, some of the 28S and 18S rRNAs were not running in the gel at the same velocity across samples, as noticed in Figs 3A and 4A.

### RNA capillary transfer

**Figure 2:**
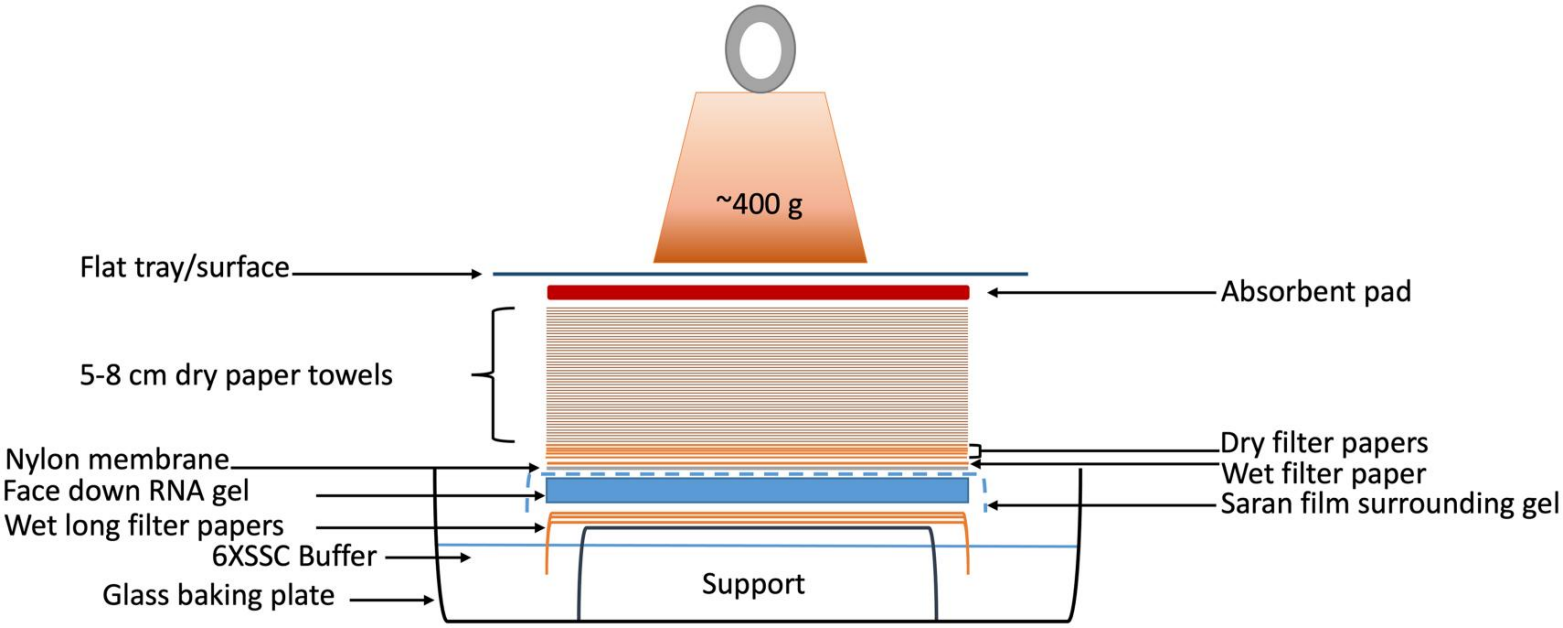
RNA capillary transfer sandwich setup. A similar setup is described in most protocols with the addition of an absorbent pad

**Figure 3:**
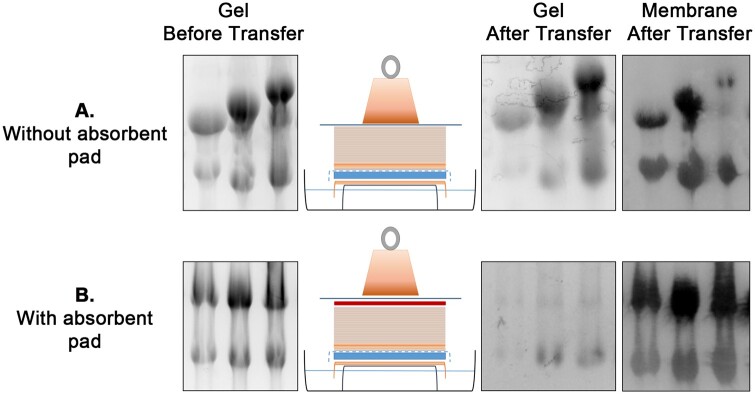
Improving the efficiency of RNA capillary transfer Note: Images of RNA gels after electrophoresis and images of gels and membranes after disassembling the transfer sandwiches were taken using the Gel Doc system. In panel (**A**), the transfer was performed without adding an absorbent pad above the layer of tissue papers. In panel (**B**), the transfer was performed with an absorbent pad addition. The upper and lower bands represent 28S and 18S rRNA, respectively. The RNA samples shown in panels (**A**) and (**B**) were isolated from HeLa cell cultures.

RNA was capillary transferred overnight at room temperature to a positively charged nylon membrane in 6× SSC at room temperature following the Nature Methods protocol [18] with indicated modifications as follows:

1. The unused parts of the RNA gel are trimmed away.
2. A small triangular corner is cut from the gel top to mark the direction of the samples after the transfer.
3. The gel is soaked in DEPC-treated water until other materials are prepared.
4. The nylon membrane was cut larger than the gel by 0.5 cm from all sides.
5. A small triangular corner was also cut from the membrane top at the same position as the gel.
6. Filter paper sheets were cut to two different sizes: 7× sheets of the same size (height to width) as the membrane and 3× sheets of a longer size (same height but larger width) to place on the top of the gel support draped over the edges to absorb the buffer from the sides (as illustrated in Fig. 2).
7. A 5–8 cm stack of paper towels was cut to the same size as the membrane.
8. The gel casting tray was used as a support by upside-down positioning in a glass baking tray.
9. About 1 l of 6× SSC transfer buffer was added to the tray containing the gel support. Note: The original protocol used 0.01 N NaOH, 3 M NaCl as a transfer buffer for the positively charged nylon membrane.
10. The three long filter paper sheets were pre-wet in the transfer buffer and were placed on the top of the gel support.
11. The gel was placed on the gel support covered by the wet filter papers in an upside-down position.
12. The gel was surrounded but not covered by parafilm or Saran wrap. A scalpel blade can be used to carefully cut a window surrounding the gel.
13. The nylon membrane was pre-wet in water and then in the 6× SSC transfer buffer.
14. The membrane was carefully placed on the top of the gel to align the cut corners. Once the membrane touches the gel, it should not be moved.
15. A gel roller or a serological pipet was used to smoothen the surface and eliminate any existing air bubbles.
16. One sheet of the seven smaller cut filter paper was pre-wet in 6× SSC and placed on the top of the membrane, aligning the cut corners. The six other filter paper sheets were placed dry on top of that.
17. The pre-cut stack of paper towels was placed on top of filter papers, and an absorbent pad was placed on top. Note: The use of an absorbent pad is optional and was not included in the original protocol. It improved the efficiency of the capillary transfer by about 80%, as illustrated in Fig. 3.
18. A flat tray or surface holding a 400-g weight was placed on the top of the sandwich.
19. The RNA transfer proceeded overnight, ensuring the weight was stable.

### RNA crosslinking

1. The transfer sandwich was disassembled the next day. Note: The transfer time was adjusted according to the user’s convenience; the original protocol restricted it to 4 h if the transfer was performed in a neutral buffer and about 1 h in the alkaline buffer.
2. The membrane was rinsed in another tray containing a clean filter paper in 2× SSC for 10–20 min, rocking at room temperature. Note: The washing in the original protocol was performed in 6× SSC for only 5 min.
3. The membrane was transferred into another tray containing a dry clean filter paper. Note: Do not allow the membrane to completely dry.
  RNA samples were auto-crosslinked to the membrane by exposures on both sides to 1200 µJ × 100 UV light using the Stratalinker 2400. Note: The original protocol mentioned several ways for RNA fixation and staining. No stain was used in the described method, and the crosslinking timing was set automatically.
4. The membrane was air-dried on another clean filter paper for 5 min. Note: This is a stop point. The membrane can be stored for weeks and wrapped in a clean plastic bag at room temperature.

### Pre-hybridization

1. Ten to fourteen milliliters of hybridization buffer was placed in a glass-capped cylinder.
2. The membrane was placed in the cylinder. Note: The air bubbles between the membrane surface and the buffer were avoided by rolling a serological pipet inside the cylinder.
3. The cylinder was stacked in a hybridization oven and kept rotating for 30 min at 37–42°C according to the used probe (37°C was used in this protocol).

### Probe preparation

All procedures described in the protocol were performed and contained in a radiation safety area in the laboratory provided with protective acrylic shields. All the radioisotope users were following the regulations and requirements of the University of Ottawa radiation safety program.

#### Short probes

Desalted probes labeled with [γ-^32^P] ATP were prepared as described in Llave *et al*. [19], briefly:

1. A mix of 12 μl nuclease-free water, 2 μl DNA oligonucleotide (10μM), 2 μl PNK buffer (10×), 2.5 μl [γ-32P] ATP, and T4 polynucleotide kinase was incubated for 45 min at 37°C.
2. The mix was run through a G25 Sephadex column and brought to 60 μl volume by nuclease-free water.
3. The probes were heated to 95°C for 5 min and snapped cold on ice.

#### Long probes

Probes were radiolabeled as described in Sambrook *et al*. [20], briefly:

1. A mix of 25 ng of clean PCR product in 30 μl nuclease-free water and 125 ng random hexamer deoxynucleotide primers was warmed for 2 min in a boiling water bath and snapped cold on ice.
2. To the mix, dCTP, dATP, dGTP, and dCTP were added (at a final concentration of 250 μM each) in addition to 10 μl of 5× random priming buffer and 5 μl [α-32P], and brought to 50 μl with nuclease-free water.
3. 5 U of Klenow DNA polymerase was added, and the reaction was incubated for 60 min at room temperature, and 10 μl stop buffer was added to terminate the reaction.
4. Labeled probes were stored at −20°C until being used.

### Hybridization and detection

1. The desalted short probes or the radiolabeled long probes were added directly to the hybridization buffer in the membrane cylinder (in the pre-hybridization section).
2. The membrane was hybridized in a containing probe overnight, rotating at 37°C. Note: Temperature varies based on the probe’s melting temperature.
3. The next day, the membrane is removed carefully from the cylinder for detection preparation. Note: The hybridization buffer containing probes can be labeled and stored correctly for further use, while the signal can be detected.
4. The membrane was washed twice in 20 ml 5× SSC for 20 min each at 37°C.
5. The membrane was washed once in 20 ml 1× SSC for 20 min at 37°C.

**Figure 4:**
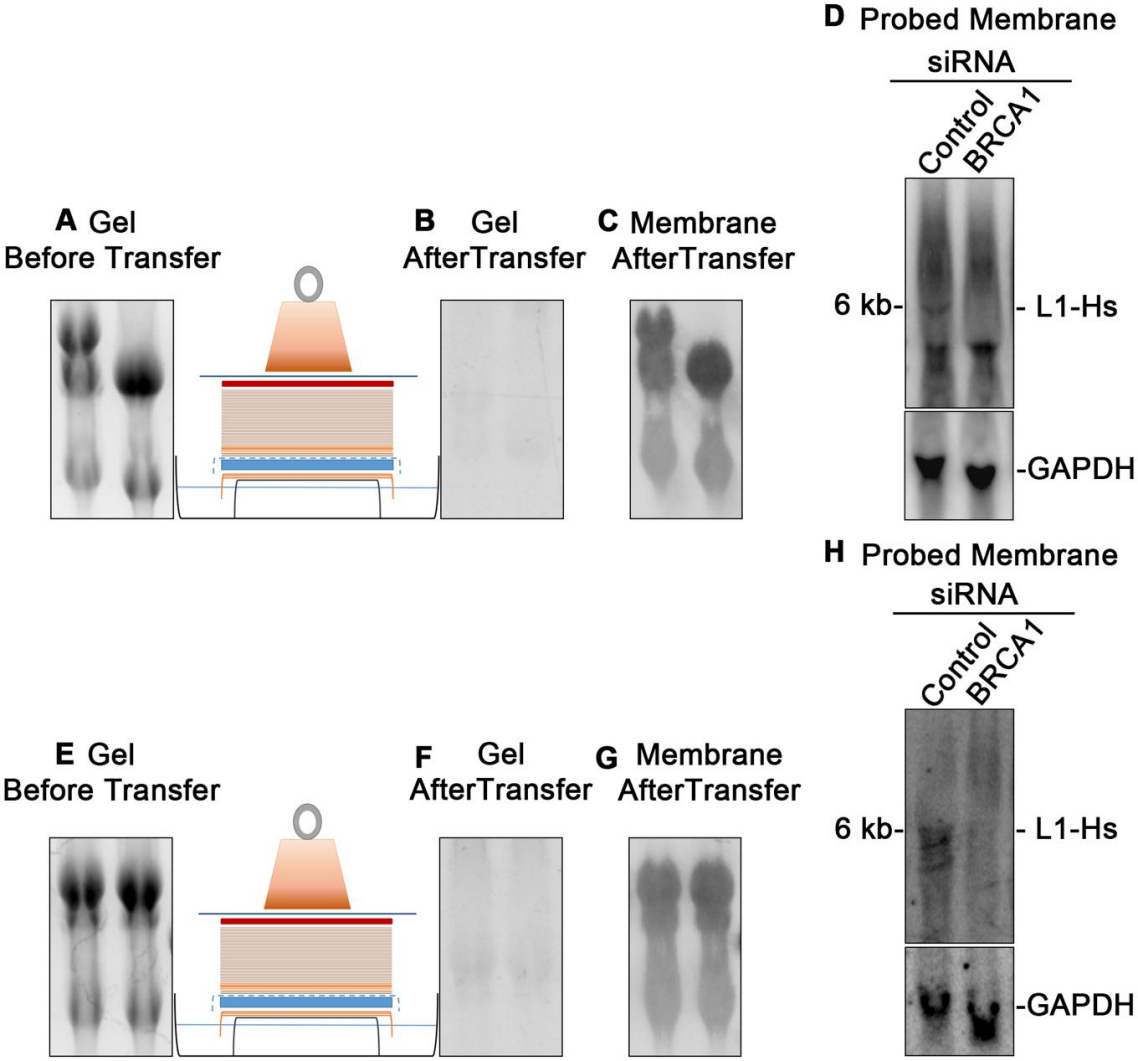
Detection of L1-Hs RNA using long and short radiolabeled probes. (**A–D**) The classical protocol that uses one long probe. (**E–H**) The new protocol that uses multiple oligonucleotide probes. (**A** and **E**) RNA gel after electrophoresis and before capillary transfer. (**B** and **F**) Gel picture after capillary transfer. (**C** and **D**) RNA is transferred efficiently to the nylon membrane after capillary transfer. (**D**) Northern blot of L1-Hs RNA from ES2 cells transfected with siRNA using long radiolabeled probes. (**H**) Northern blot of L1-Hs RNA from ES2 cells transfected with siRNA using multiple short, radiolabeled probes.

A phosphorimager or X-ray films were used to detect the signal (Fig. 4). Note: Typhoon screen was more sensitive than films supported by an intensifying screen.

### Stripping and re-probing

The membranes could be re-used up to 3× using the steps below:

1. 1× SCC buffer was boiled using an electric kettle.
2. The membrane was placed in a small tray and covered with excess boiled buffer.
3. The membrane was incubated, rocking for 5–10 min at room temperature until the buffer was cooled down.
4. Steps 2 and 3 were repeated once more.
5. The membrane was washed in 2× SSC once and proceeded to the pre-hybridization steps. Note: Stripping efficiency is confirmed by losing the previously seen signal upon detection.

## Results and discussion

The above-described method was used to validate the impact of BRCA1 on the full-length competent L1-Hs. By depleting the cells of BRCA1, the expression of the 6 kb L1 RNA was reduced (Fig. 4D and H). In the author’s previous work, BRCA1 was demonstrated in various cell models to directly regulate the transcription of full-length L1-Hs elements [15]. Since our interest was to detect the full-length RNA derived by the L1 sense promoter, the long probe was prepared similarly to the classical approach described in Deininger *et al.* [11] but designed to be specific to the 5′ first 486 bp of the L1-Hs. A non-specific background was detected using this approach (Fig. 4D), presumably related to the spliced and prematurely polyadenylated RNAs detected in Deininger *et al.* [11]. In our alternative new protocol to blot for the competent L1-Hs RNA, we aligned the consensus sequences of L1-PA and L1-Hs and designed a dozen short probes at areas of mismatches between the two sequences in the 5′-UTR (1–900 bp). Combining the 12 probes improved the specificity of the blot by reducing the background bands noticed using the classical approach. Adding the Poly A selection step to classical and new protocols described in this method may enhance the signal-to-noise ratio and overcome the resulting blot quality limitation. The described alternative method of combining multiple short probes is suitable to use if the target RNA sequence is previously known not for investigating a novel RNA, which limits its applicability.

Besides showing that detecting active L1-Hs RNA is possible using alternative means, the described protocol improved the general capillary transfer step necessary for nucleic acid blotting (northern and Southern). Although advanced sequencing techniques have replaced these blotting techniques to study RNA and DNA regulation, the bioinformatic analysis of retrotransposons remains challenging. The repetitive nature of retrotransposons and their inclusion in other genes complicate interpreting the data investigating authentic and specific elements with an increased signal-to-noise ratio.

Despite the capability of detecting endogenous L1-Hs RNA, this method is limited by the small number of full-length elements available to blot in the genome. This limitation resulted in medium-quality blots. It is also restricted to testing in cell lines; validating the method in cells overexpressing L1 in addition to different tissues (as described in Belancio *et al.* [9]) would be attractive for prospective work. The described method, combined with tips and tricks from various cited northern blotting protocols, can be applied to study the endogenous expression of human-specific competent L1 RNA. In addition, the improved protocol of the capillary transfer technique can serve a wide range of scientists still using blotting techniques.

## Conclusions

This article has described a methodology to design northern blotting probes specifically detecting active full-length L1-Hs following two approaches: classical using a long probe and a new alternative using multiple short probes. The protocol also enclosed tips and tricks for an enhanced RNA transfer using the standard capillary technique, which can help blot all nucleic acids. This method is limited by the low number of blotted elements in the tested specimens, the quality of gel running, and RNA detection, which can be improved in future applications.

## Supplementary Material

bpae036_Supplementary_Data

## Data Availability

Not applicable.
