# Supplementary material for: Northern blotting of endogenous full-length human-specific LINE-1 RNA
Source: Biol Methods Protoc. 2024 May 28;9(1):bpae036. doi: 10.1093/biomethods/bpae036 (PMC11320832; doi:10.1093/biomethods/bpae036)
Supplement: bpae036_Supplementary_Data [file bpae036_supplementary_data.zip › Revised Appendix.pdf]

Appendix for

Northern blotting of endogenous full-length human-specific LINE-1 RNA

This PDF file contains:

Section 1: Classical Approach	Page 2
Section 2: Alternative Approach	Pages 3-4
Section 3: Sequence alignment and mismatches of short probes with L1PA subfamilies	Pages 5-11

Section 1: Classical Approach

Green text and arrows denote the start and end of amplified regions by using a long RNA probe in L1-Hs versus L1-PA2 in CLUSTAL O (1.2.4) multiple sequence alignment.

Red characters denote mismatch (between aligned L1-Hs and L1-PA2) locations in the L1-PA2 Repbase consensus sequence.

```
L1-HS-Repbase       gggaggaggagccaagatggccgaataggaacagctccggtctacagctcccagcgtgag    60
L1-PA2-Repbase      -gggggaggagccaagatggccgaataggaacagctccggtctacagctcccagcgtgag    59
                     ** ********************************************************

L1-HS-Repbase       cgacgcagaagacgggtgatttctgcatttccatctgaggtaccgggttcatctcactag    120
L1-PA2-Repbase      cgacgcagaagacgggtgatttctgcatttccaactgaggtaccaggttcatctcactgg    119
                    ********************************* ********** ************* *

L1-HS-Repbase       ggagtgccagacagtgggcgcaggccagtgtgtg-tgcgcaccgtgcgcgagccgaagca    179
L1-PA2-Repbase      ggagtgccagacagtgggcgcaggacagtgggtgcagcgcaccgtgcgtgagccgaagca    179
                    ************************ ***** ***  ************ ***********

L1-HS-Repbase       gggcgaggcattgcctcacctgggaagcgcaaggggtcagggagttccctttccgagtca    239
L1-PA2-Repbase      gggcgaggcatcgcctcacccgggaagcgcaaggggtcagggaattccctttcctagtca    239
                    *********** ******** ********************** ********** *****

L1-HS-Repbase       aagaaaggggtgacggac-gcacctggaaaatcgggtcactcccacccgaatattgcgct    298
L1-PA2-Repbase      aagaaaggggtgacagacggcacctggaaaatcgggtcactcccgccctaatactgcgct    299
                    ************** *** ************************* *** **** ******

L1-HS-Repbase       tttcagaccggcttaagaaacggcgcaccacgagactatatcccacacctggctcagagg    358
L1-PA2-Repbase      tttccgacgggcttaaaaaacggcgcaccaggagattatatcccgcacctggctcggagg    359
                    **** *** ******* ************* **** ******** ********** ****

L1-HS-Repbase       gtcctacgcccacggaatctcgctgattgctagcacagcagtctgagatcaaactgcaag    418
L1-PA2-Repbase      gtcctacgcccacggagtctcgctgattgctagcacagcagtccgagatcaaactgcaag    419
                    **************** ************************** ****************

L1-HS-Repbase       gcggcaacgaggctgggggaggggcgcccgccattgcccaggcttgcttaggtaaacaaa    478
L1-PA2-Repbase      gcggcagcgaggctgggggaggggcgcccgccattgcccaggcttgattaggtaaacaaa    479
                    ****** *************************************** *************

L1-HS-Repbase       gcagccgggaagctcgaactgggtggagcccaccacagctcaaggaggcctgcctgcctc    538
L1-PA2-Repbase      gcggccgggaagctcgaactgggtggagcccaccacagctcaaggaggcctgcctgcctc    539
                    ** *********************************************************
```

Section 2: Alternative Approach

Blue text and boxes denote the short RNA probes (P1-P12) localized in the L1-Hs Repbase consensus sequence displayed in CLUSTAL O (1.2.4) multiple sequence alignment.

Red characters denote mismatch (between aligned L1-Hs and L1-PA2) locations in the L1-PA2 Repbase consensus sequence.

```
L1-HS-Repbase       gggaggaggagccaagatggccgaataggaacagctccggtctacagctcccagcgtgag    60
L1-PA2-Repbase      -ggggaggagccaagatggccgaataggaacagctccggtctacagctcccagcgtgag    59
                     ** ********************************************************

                                                                              P1
L1-HS-Repbase       cgacgcagaagacgggtgatttctgcatttccatctgaggtaccgggttcatctcactag    120
L1-PA2-Repbase      cgacgcagaagacgggtgatttctgcatttccaactgaggtaccaggttcatctcactgg    119
                    ********************************* ********** ************* *

L1-HS-Repbase       ggagtgccagacagtgggcgcaggccagtgtgtg-tgcgcaccgtgcgcgagccgaagca    179
L1-PA2-Repbase      ggagtgccagacagtgggcgcaggacagtgggtgcagcgcaccgtgcgtgagccgaagca    179
                    ************************ ***** ***  ************ ***********

                                                                              P2
L1-HS-Repbase       gggcgaggcattgcctcacctgggaagcgcaaggggtcagggagttccctttccgagtca    239
L1-PA2-Repbase      gggcgaggcatcgcctcacccgggaagcgcaaggggtcagggaattccctttcctagtca    239
                    *********** ******** ********************** ********** *****

                             P3                                 P9
L1-HS-Repbase       aagaaaggggtgacggac-gcacctggaaaatcgggtcactcccacccgaatattgcgct    298
L1-PA2-Repbase      aagaaaggggtgacagacggcacctggaaaatcgggtcactcccgccctaatactgcgct    299
                    ************** *** ************************* *** **** ******

                                         P10                   P11
L1-HS-Repbase       tttcagaccggcttaagaaacggcgcaccacgagactatatcccacacctggctcagagg    358
L1-PA2-Repbase      tttccgacgggcttaaaaacggcgcaccaggagattatatcccgcacctggctcggagg    359
                    **** *** ******* ************* **** ******** ********** ****

L1-HS-Repbase       gtcctacgcccacggaatctcgctgattgctagcacagcagtctgagatcaaactgcaag    418
L1-PA2-Repbase      gtcctacgcccacggagtctcgctgattgctagcacagcagtccgagatcaaactgcaag    419
                    **************** ************************* *****************

                                                                              P4
L1-HS-Repbase       gcggcaacgaggctgggggagggcgcccgccattgcccaggcttgcttaggtaaacaaa    478
L1-PA2-Repbase      gcggcagcgaggctgggggagggcgcccgccattgcccaggcttgattaggtaaacaaa    479
                    ****** ************************************** **************

L1-HS-Repbase       gcagccgggaagctcgaactgggtggagcccaccacagctcaaggaggcctgcctgcctc    538
L1-PA2-Repbase      gcggccgggaagctcgaactgggtggagcccaccacagctcaaggaggcctgcctgcctc    539
                    ** *********************************************************

L1-HS-Repbase       tgtaggctccacctctgggggcagggcacagacaaacaaaaagacagcagtaacctctgc    598
L1-PA2-Repbase      tgtaggctccacctctgggggcagggcacagacaaacaaaaagacagcagtaacctctgc    599
                    ************************************************************

L1-HS-Repbase       agacttaagtgtccctgtctgacagctttgaagagagcagtggttctcccagcacgcagc    658
L1-PA2-Repbase      agacttaaatgtccctgtctgacagctttgaagagagcagtggttctcccagcacgcagc    659
                    ******** ***************************************************

                                                                         P5
L1-HS-Repbase       tggagatctgagaacgggcagactgcctcctcaagtgggtccctgacccctgacccccga    718
L1-PA2-Repbase      ttcagatctgagaacgggcagactgcctcctcaagtgggtccctgaccccgagtagcc-    718
                    *  *********************************************** **    *

                 P7                                 P8
L1-HS-Repbase       gcagcctaactgggaggcaccccccagcaggggcacactgaca-----------------    761
L1-PA2-Repbase      ------taactgggaggcacccccccagtaggggcggactgacacctcacacggccgggta    772
                          ********************* ******  *******
```

```
L1-HS-Repbase    ------------------------------------------------------------    761
L1-PA2-Repbase   ctcctctgagacaaaacttccagaggaacgatcaggcagcagcatctgcggttcaccaat    832

                                                                             P12
L1-HS-Repbase    ---------------------------------------------------cctcacacg    770
L1-PA2-Repbase   atccactgttctgcagccaccgctgctgatacccaggcaaacagggtctggagtggacct    892
                                                                      *    *

L1-HS-Repbase    gcagggtattccaacagacctgcagctgagggtcctgtctgttagaaggaaaactaacaa    830
L1-PA2-Repbase   ccagcaaactccaacagacctgcagctgagggtcctgtctgttagaaggaaaactaacaa    952
                  ***   * **************************************************

                                                           P6
L1-HS-Repbase    ccagaaaggacatctacaccgaaaacccatctgtacatcaccatcatcaaagaccaaaag    890
L1-PA2-Repbase   acagaaaggacatccacaccaaaaacccatctgtacgtcaccatcatcaaagaccaaagg    1012
                  ************* ***** *************** ********************* *

L1-HS-Repbase    tagataaaaccacaaagatggggaaaaaacagaacagaaaaactggaaactctaaaacgc    950
L1-PA2-Repbase   tagataaaaccacaaagatggggaaaaaacagagcagaaaaactggaaactctaaaaatc    1072
                 ********************************* ***********************  *
```

Section 3: Sequence alignment and mismatches of short probes with L1PA subfamilies

Blue text and boxes denote the short RNA probes (P1-P12) aligned with the L1-Hs Repbase consensus sequence (in black font) and DFAM sequences of L1PA subfamilies (in gray font) using CLUSTAL O (1.2.4).

Red characters denote mismatches (between aligned L1-Hs and L1-PA) locations in the L1-PA DFAM consensus sequences.

```
DFAM-L1PA17_5end          tggggcgcagaaaaggaaagcagcaagagtctggcagagatcgacccccgaggaactcgg
        205
DFAM-L1PA15-16_5end       ---acgcgggcaaacagcccccgtgacg--------gcgtccggctgataaaagtgagtg
        209
DFAM-L1PA12_5end          agggcggggcgacggcccacccgggagc--------gacacgga--gccaagggaacctc
        212
DFAM-L1PA10_5end          agggtggggcgncgcctcacccgggagg--------cgcanggg--gcnagggaactcc
        216
L1PA2-Repbase             agggcgaggcatcgcctcacccgggaag--------cgcaaggg--gtcagggaattccc
        228
L1HS-Repbase              agggcgaggcattgcctcacctgggaag--------cgcaaggg--gtcagggagttccc
        228
L1-HS-5UTR-P12            ------------------------------------------------------------
        0
L1-HS-5UTR-P11            ------------------------------------------------------------
        0
L1-HS-5UTR-P10            ------------------------------------------------------------
        0
L1-HS-5UTR-P9             ------------------------------------------------------------
        0
L1-HS-5UTR-P8             ------------------------------------------------------------
        0
L1-HS-5UTR-P7             ------------------------------------------------------------
        0
L1-HS-5UTR-P6             ------------------------------------------------------------
        0
L1-HS-5UTR-P5             ------------------------------------------------------------
        0
L1-HS-5UTR-P4             ------------------------------------------------------------
        0
L1-HS-5UTR-P3             ------------------------------------------------------------
        0
L1-HS-5UTR-P2             ----------------------------------------------------GAGTTCCC
        8
L1-HS-5UTR-P1             ------------------------------------------------------------
        22

DFAM-L1PA13_5end          gc-cccc-agcgact--ccgggggaacgggtgagttgaactggcaaggagcaacccgctc
        258
DFAM-L1PA17_5end          agccccgcggaaa------gggtaggtggggtgcttctctgctcccctcaccccc----t
        255
DFAM-L1PA15-16_5end       aagccccagt----------acgtgagagaggcagagagcctccctctgtgactcaccttt
        260
DFAM-L1PA12_5end          cccc----gcccagggaagcggtg----agtgaatgtgcgaccccgggaaaccac-gctt
        263
DFAM-L1PA10_5end          cnccccccagccaagggaagccgtgagngactgtgctacccngcccggganactac-gctt
        275
L1PA2-Repbase             tt-tcctagtcaaagaaaggggtgacagacggcacctggaaaatcgggtcactcc-----
        282
```

```
L1HS-Repbase            tt-tccgagtcaaagaaaggggtgacggac-gcacctggaaaatcgggtcactcc-----
        281
L1-HS-5UTR-P12          ------------------------------------------------------------
        0
L1-HS-5UTR-P11          ------------------------------------------------------------
        0
L1-HS-5UTR-P10          ------------------------------------------------------------
        0
L1-HS-5UTR-P9           -----------------------------------------------GTCACTCC-----
        8
L1-HS-5UTR-P8           ------------------------------------------------------------
        0
L1-HS-5UTR-P7           ------------------------------------------------------------
        0
L1-HS-5UTR-P6           ------------------------------------------------------------
        0
L1-HS-5UTR-P5           ------------------------------------------------------------
        0
L1-HS-5UTR-P4           ------------------------------------------------------------
        0
L1-HS-5UTR-P3           -------------------------CGGAC-GCACCTGGAAAATCG--------------
        20
L1-HS-5UTR-P2           TT-TCCGAGTCAAAG---------------------------------------------
        22
L1-HS-5UTR-P1           ------------------------------------------------------------
        22


DFAM-L1PA13_5end        tcgccacgggcctctggaatcccggcaggaggagacccct--cgaccaccacg-------
        309
DFAM-L1PA17_5end        gcgacaanctgctgaccgccaaa--ctgtcgggagccccc----tctgccctcgcg----
        305
DFAM-L1PA15-16_5end     ccactggggatccgagcaacccaggccgagggagagcactttgtttctcccaa-------
        313
DFAM-L1PA12_5end        ctcccacggatctttgcaaccctcgggtcaggagatcccc-tcgtgagcccactccacca
        322
DFAM-L1PA10_5end        ttcccacggatcttcgcaacccgcggaccaggagatccccctcgtgngcccacgccacca
        335
L1PA2-Repbase           -------------------------cgccctaatactgcgcttttccgacggg-------
        310
L1HS-Repbase            -------------------------cacccgaatattgcgcttttcagaccgg-------
        309
L1-HS-5UTR-P12          ------------------------------------------------------------
        0
L1-HS-5UTR-P11          ------------------------------------------------------------
        0
L1-HS-5UTR-P10          --------------------------------------------TCAGACCGG-------
        9
L1-HS-5UTR-P9           -------------------------CACCCGAATATTGC---------------------
        22
L1-HS-5UTR-P8           ------------------------------------------------------------
        0
L1-HS-5UTR-P7           ------------------------------------------------------------
        0
L1-HS-5UTR-P6           ------------------------------------------------------------
        0
L1-HS-5UTR-P5           ------------------------------------------------------------
        0
L1-HS-5UTR-P4           ------------------------------------------------------------
        0
```

```
L1-HS-5UTR-P3               ------------------------------------------------------------
        20
L1-HS-5UTR-P2               ------------------------------------------------------------
        22
L1-HS-5UTR-P1               ------------------------------------------------------------
        22


DFAM-L1PA13_5end            -gacacttgagttggcagggagagctgcttagagaagtggtaggggc-------------
         355
DFAM-L1PA17_5end            --ancccgggcaacgctgtcggtggcgatttgggaacttcccgggga------------c
         351
DFAM-L1PA15-16_5end         --gccctggagctaacttggggagaggcttggagacgctgtgagggaaagacaccgggaa
         371
DFAM-L1PA12_5end            gggccttcggtctgacacacagagctgcgtgga-gtctcggcagagc---agccgctcag
         378
DFAM-L1PA10_5end            gggccctgggtcccaagcacagagct-gtgcag-attctcagcggcc---actcggctgg
         390
L1PA2-Repbase               ----------cttaaaaaacggcgcaccaggag-attatatcccgca----cctggctcg
         355
L1HS-Repbase                ----------cttaagaaacggcgcaccacgag-actatatcccaca----cctggctca
         354
L1-HS-5UTR-P12              ------------------------------------------------------------
        0
L1-HS-5UTR-P11              ---------------------------CACGAG-ACTATATCCCACA----CCT------
        22
L1-HS-5UTR-P10              ----------CTTAAGAAACGGC-------------------------------------
        22
L1-HS-5UTR-P9               ------------------------------------------------------------
        22
L1-HS-5UTR-P8               ------------------------------------------------------------
        0
L1-HS-5UTR-P7               ------------------------------------------------------------
        0
L1-HS-5UTR-P6               ------------------------------------------------------------
        0
L1-HS-5UTR-P5               ------------------------------------------------------------
        0
L1-HS-5UTR-P4               ------------------------------------------------------------
        0
L1-HS-5UTR-P3               ------------------------------------------------------------
        20
L1-HS-5UTR-P2               ------------------------------------------------------------
        22
L1-HS-5UTR-P1               ------------------------------------------------------------
        22


DFAM-L1PA13_5end            agagggtttggtgcgggagcgtctgtagcgg------------------agcacggccag
         421
DFAM-L1PA17_5end            acccgttgtg---------ggccac-tgccctgcccggggaacctcagcccttgngtcnc
         486
DFAM-L1PA15-16_5end         aggcattttagtc---tcgggccag---agattggagcgcctgctctggagcggggtagg
         532
DFAM-L1PA12_5end            gtacatacccctaggaagggggctg--aatccagggggccgagcagcgncggtctgcggg
         532
DFAM-L1PA10_5end            tccccacagcgcagcanagcggctgtggcagatcgtggccagactgcctctctaggccgg
         569
L1PA2-Repbase               ccgccattgcccaggcttgattaggtaaacaaagcggccgggaagctcgaactgggt-gg
         505
```

```
L1HS-Repbase                ccgccattgcccaggcttgcttaggtaaacaaagcagccgggaagctcgaactgggt-gg
        504
L1-HS-5UTR-P12              ------------------------------------------------------------
        0
L1-HS-5UTR-P11              ------------------------------------------------------------
        22
L1-HS-5UTR-P10              ------------------------------------------------------------
        22
L1-HS-5UTR-P9               ------------------------------------------------------------
        22
L1-HS-5UTR-P8               ------------------------------------------------------------
        0
L1-HS-5UTR-P7               ------------------------------------------------------------
        0
L1-HS-5UTR-P6               ------------------------------------------------------------
        0
L1-HS-5UTR-P5               ------------------------------------------------------------
        0
L1-HS-5UTR-P4               ------------------GCTTAGGTAAACAAAGCAGCCG--------------------
        22
L1-HS-5UTR-P3               ------------------------------------------------------------
        20
L1-HS-5UTR-P2               ------------------------------------------------------------
        22
L1-HS-5UTR-P1               ------------------------------------------------------------
        22


DFAM-L1PA13_5end            gccctgggggcccgcatcatagctcctgcgctggcg--gaccgtgcctgaccggcggaga
        668
DFAM-L1PA17_5end            acaaaggaaacgcgggcgcggcgccaaccgctgaaggggcagcaccgacgcccgggaac
        713
DFAM-L1PA15-16_5end         gacctggaaccggtctgcgtgtgccat-tgctgggt--gccccagcctgctcccctgaga
        736
DFAM-L1PA12_5end            taccaaaacgtggccagactgcttctttaagcgggn--ccccgatcccgttcctc-----
        803
DFAM-L1PA10_5end            cgccaagggacagncagantgcctcctcaagtgggt--ccctgaccccgtgcctc-----
        848
L1PA2-Repbase               -atctgagaacgggcagactgcctcctcaagtgggt--ccctgaccccgagtagcc---
        718
L1HS-Repbase                -atctgagaacgggcagactgcctcctcaagtgggt--ccctgacccctgacccccgagc
        720
L1-HS-5UTR-P12              ------------------------------------------------------------
        0
L1-HS-5UTR-P11              ------------------------------------------------------------
        22
L1-HS-5UTR-P10              ------------------------------------------------------------
        22
L1-HS-5UTR-P9               ------------------------------------------------------------
        22
L1-HS-5UTR-P8               ------------------------------------------------------------
        0
L1-HS-5UTR-P7               -----------------------------------------------------------C
        1
L1-HS-5UTR-P6               ------------------------------------------------------------
        0
L1-HS-5UTR-P5               -------------------------------TGGGT--CCCTGACCCCTGACC-------
        20
L1-HS-5UTR-P4               ------------------------------------------------------------
        22
L1-HS-5UTR-P3               ------------------------------------------------------------
        20
```

L1-HS-5UTR-P2 --
22
L1-HS-5UTR-P1 --
22

DFAM-L1PA13_5end gc---tccagcggggcggcccccacgg--ccacgcaccagcccgcc----cgctccctcc
719
DFAM-L1PA17_5end ggacgtggagaggggggtcatctcccgc-tccccccgtccactgttgcggacgcagcagcg
772
DFAM-L1PA15-16_5end tc--gtggtgcagcggggcctctccgctccacgcccaggcagatc---------------
780
DFAM-L1PA12_5end -----ctcactgggcgggacctcccaa--c-cggggcctccagcca--------------
841
DFAM-L1PA10_5end -----ctgactgggngagacctcccaa--c-aggggtcgncagaca--------------
886
L1PA2-Repbase ------taactgggaggcaccccccag--t-aggggcggactgaca--------------
755
L1HS-Repbase **ag--cctaactgggaggcaccccccag--c-aggggcacactgaca--------------**
761
L1-HS-5UTR-P12 --
0
L1-HS-5UTR-P11 --
22
L1-HS-5UTR-P10 --
22
L1-HS-5UTR-P9 --
22
L1-HS-5UTR-P8 -----------------------CCAG--C-AGGGGCACACTGACA--------------
20
L1-HS-5UTR-P7 AG--CCTAACTGGGAGGCACC---------------------------------------
20
L1-HS-5UTR-P6 --
0
L1-HS-5UTR-P5 --
20
L1-HS-5UTR-P4 --
22
L1-HS-5UTR-P3 --
20
L1-HS-5UTR-P2 --
22
L1-HS-5UTR-P1 --
22

DFAM-L1PA13_5end gcgtgtgcgcgtgcaccctgccctgccactgctgcggcgngagtgcactccgccccccct
878
DFAM-L1PA17_5end gaagggcggggcccanctccccctccc-tacacagagcggcagcgtcccngcaacgga-g
941
DFAM-L1PA15-16_5end gcattcggagcacccgctc---------g------------cctggatcagcagcctg--
935
DFAM-L1PA12_5end gctttggagagtccaagcc---------gaccgggggcggaagcggtcccccagcaca-g
1009
DFAM-L1PA10_5end gcgcgggagnganccaggc---------gaacagggtctggagtggacccccagcaaa-c
1055
L1PA2-Repbase ccgctgctgatacccaggc---------aaacagggtctggagtggacctccagcaaa-c
901
L1HS-Repbase **---cctcacacggcagggta-t**
779

```
L1-HS-5UTR-P12          ------------------------------------------CTCACACGGCAGGGTA-T
        17
L1-HS-5UTR-P11          ------------------------------------------------------------
        22
L1-HS-5UTR-P10          ------------------------------------------------------------
        22
L1-HS-5UTR-P9           ------------------------------------------------------------
        22
L1-HS-5UTR-P8           ------------------------------------------------------------
        20
L1-HS-5UTR-P7           ------------------------------------------------------------
        20
L1-HS-5UTR-P6           ------------------------------------------------------------
        0
L1-HS-5UTR-P5           ------------------------------------------------------------
        20
L1-HS-5UTR-P4           ------------------------------------------------------------
        22
L1-HS-5UTR-P3           ------------------------------------------------------------
        20
L1-HS-5UTR-P2           ------------------------------------------------------------
        22
L1-HS-5UTR-P1           ------------------------------------------------------------
        22


DFAM-L1PA13_5end        cccccgccgcaccgccattgcagtcggagccttggcgggcacagagccngccag-----
        933
DFAM-L1PA17_5end        gacagacgagccacagagctgtctgctctggactgggggaagagg---------------
        986
DFAM-L1PA15-16_5end     -------------------agccgccccacccttcctgtgcagagatcntggtgcagcgg
        976
DFAM-L1PA12_5end        cacagctgctctacgaaaacgtggccagactgcttctttaagcgggtccccga-------
        1062
DFAM-L1PA10_5end        cgcagcagncctgcagaagaggggcctgactgttagaagaaaaacnaacaaacagaaagc
        1115
L1PA2-Repbase           tccaacagacctgcagctgagggtcctgtctgttagaaggaaaactaacaaacagaaagg
        961
L1HS-Repbase            tccaacagacctgcagctgagggtcctgtctgttagaaggaaaactaacaaccagaaagg
        839
L1-HS-5UTR-P12          TCCAA-------------------------------------------------------
        22
L1-HS-5UTR-P11          ------------------------------------------------------------
        22
L1-HS-5UTR-P10          ------------------------------------------------------------
        22
L1-HS-5UTR-P9           ------------------------------------------------------------
        22
L1-HS-5UTR-P8           ------------------------------------------------------------
        20
L1-HS-5UTR-P7           ------------------------------------------------------------
        20
L1-HS-5UTR-P6           ------------------------------------------------------------
        0
L1-HS-5UTR-P5           ------------------------------------------------------------
        20
L1-HS-5UTR-P4           ------------------------------------------------------------
        22
L1-HS-5UTR-P3           ------------------------------------------------------------
        20
```

```
L1-HS-5UTR-P2          ------------------------------------------------------------
        22
L1-HS-5UTR-P1          ------------------------------------------------------------
        22


DFAM-L1PA13_5end       ---------ccccgccccgccagcgccccg--------cccctgcgccaacactgccgcc
        977
DFAM-L1PA17_5end       ---------------------------------------ctctgccccgagcccatttcg
        1007
DFAM-L1PA15-16_5end    ggccctctccgctccacgcccaggcagatct---ccaggcattcggagcacccgctcgcc
        1033
DFAM-L1PA12_5end       -tcccgttcctcctcactgggcgggacctcccaaccggggcctccagccac-ccccgccg
        1120
DFAM-L1PA10_5end       aacaacancaacatcaacaaaaaggnccccacaaaaacccatccaa-----aggtcanc
        1170
L1PA2-Repbase          acat----------------------ccacaccaaaaacccatctgt-----acgtcacc
        994
L1HS-Repbase           acat----------------------ctacaccgaaaacccatctgt-----acatcacc
        872
L1-HS-5UTR-P12         ------------------------------------------------------------
        22
L1-HS-5UTR-P11         ------------------------------------------------------------
        22
L1-HS-5UTR-P10         ------------------------------------------------------------
        22
L1-HS-5UTR-P9          ------------------------------------------------------------
        22
L1-HS-5UTR-P8          ------------------------------------------------------------
        20
L1-HS-5UTR-P7          ------------------------------------------------------------
        20
L1-HS-5UTR-P6          ------------------------------CCGAAAACCCATCTGT-----ACATCA--
        22
L1-HS-5UTR-P5          ------------------------------------------------------------
        20
L1-HS-5UTR-P4          ------------------------------------------------------------
        22
L1-HS-5UTR-P3          ------------------------------------------------------------
        20
L1-HS-5UTR-P2          ------------------------------------------------------------
        22
L1-HS-5UTR-P1          ------------------------------------------------------------
        22
```
