# Supplementary material for: Northern blotting of endogenous full-length human-specific LINE-1 RNA
Source: Biol Methods Protoc. 2024 May 28;9(1):bpae036. doi: 10.1093/biomethods/bpae036 (PMC11320832; doi:10.1093/biomethods/bpae036)

Supplementary Data

Northern blotting of endogenous full-length human-specific LINE-1 RNA

This PDF file contains the full blot images :

Figure 3A	Page1
Figure 3B	Page2
Figure 4A-C	Page 3
Figure 4D	Page 4
Figure 4E-G	Page 5
Figure 4H	Page 6

Figure 3A

- RNA isolated from HeLa cells

Gel Before Capillary Transfer

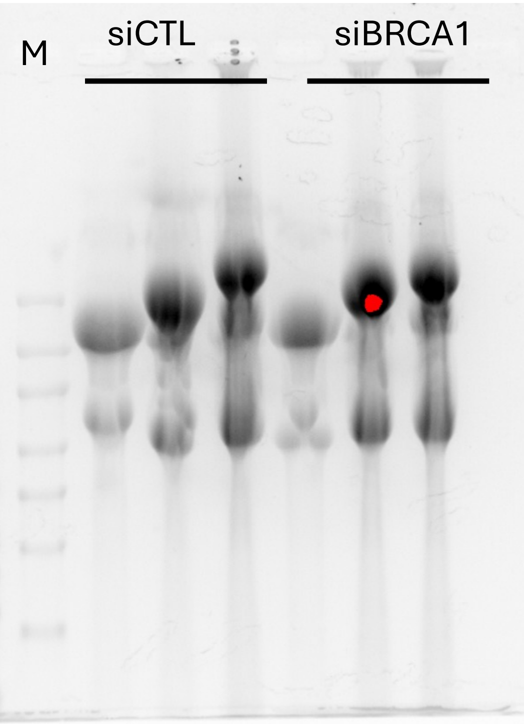

Gel After Capillary Transfer

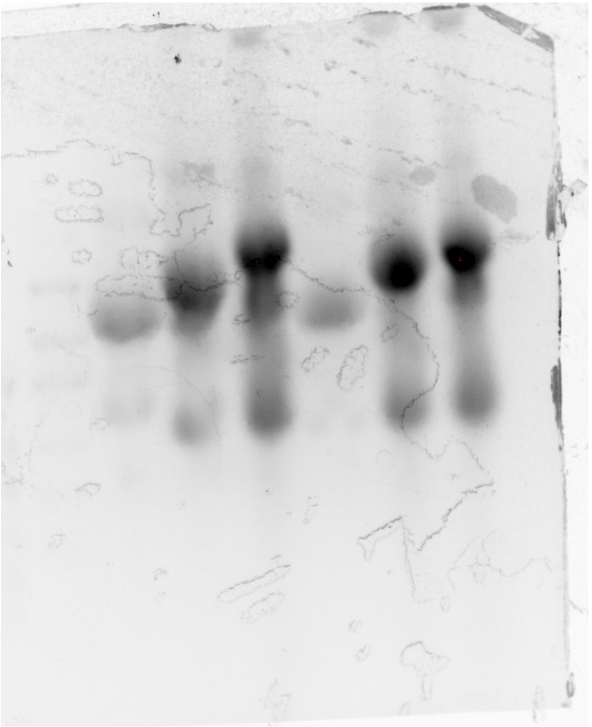

Membrane After Capillary Transfer

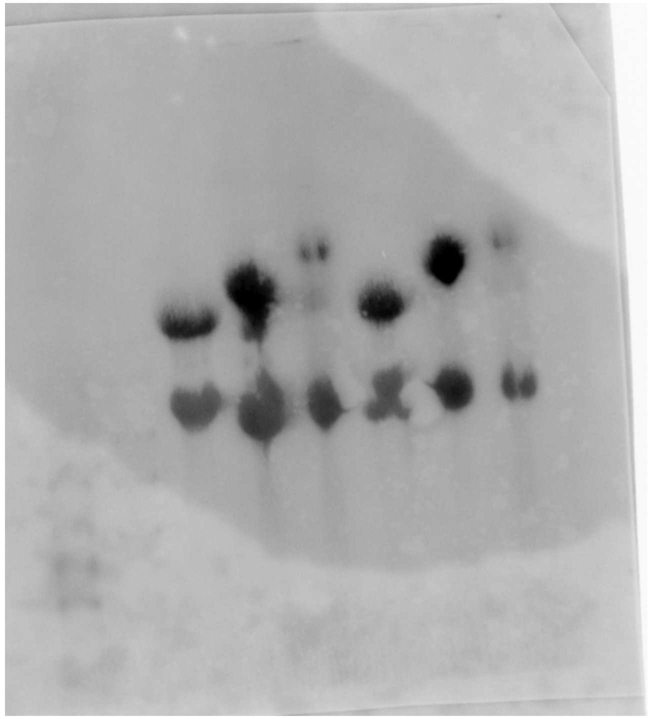

Figure 3B

- RNA isolated from HeLa cells

Gel Before Capillary Transfer

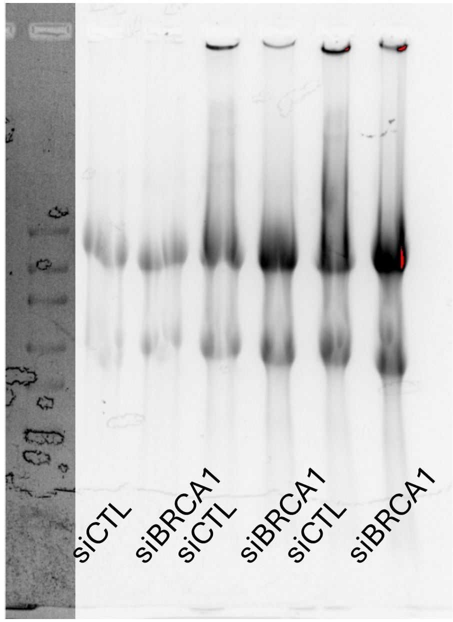

Gel After Capillary Transfer

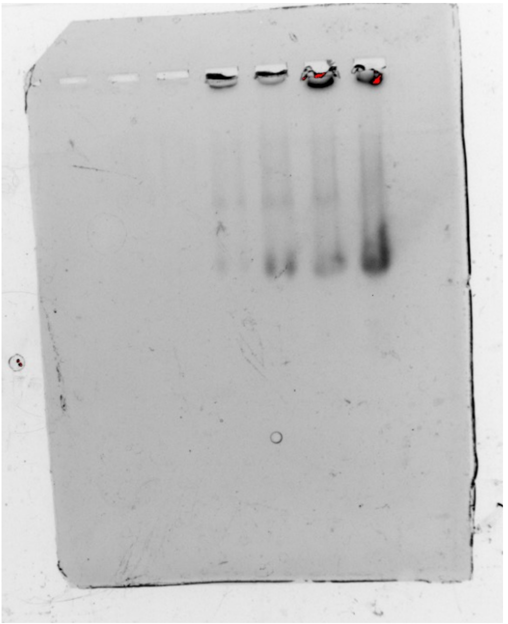

Membrane After Capillary Transfer

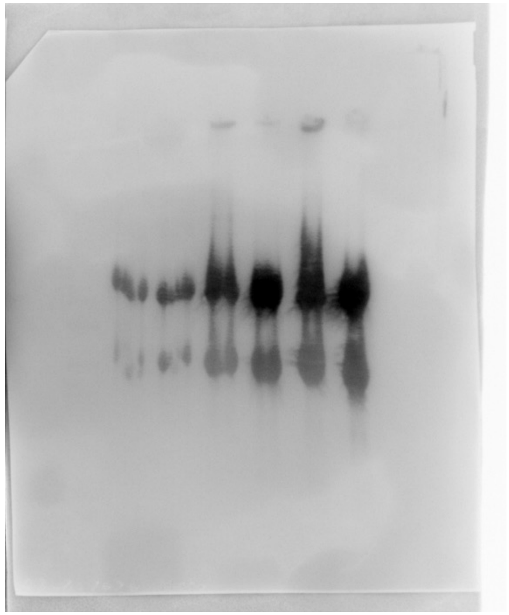

Figure 4A-C

- RNA isolated from ES2 cells

Gel Before Capillary Transfer

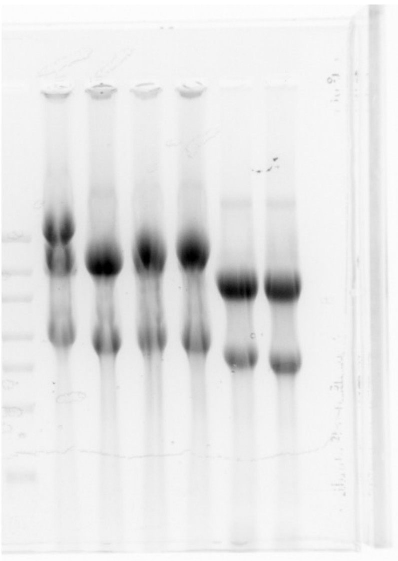

Gel After Capillary Transfer

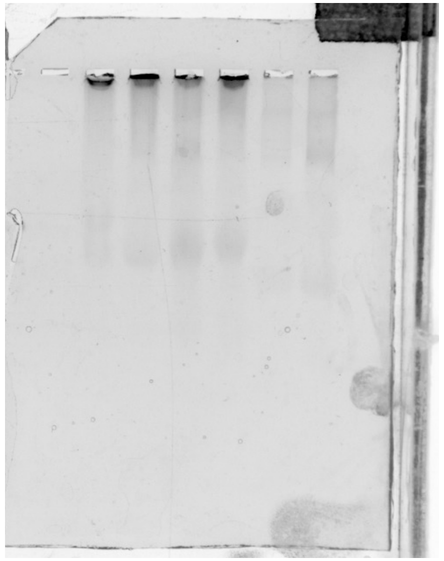

Membrane After Capillary Transfer

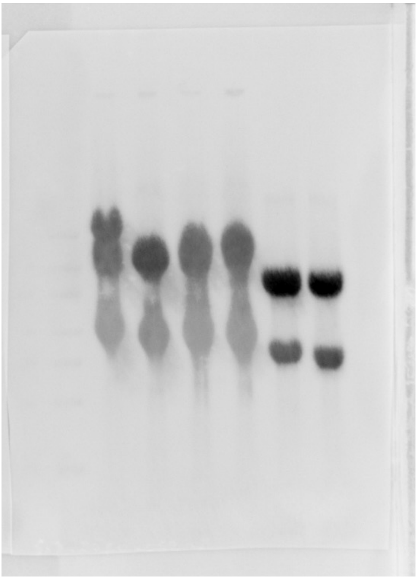

Figure 4D

- Probed membrane with L1-Hs using the classical approach

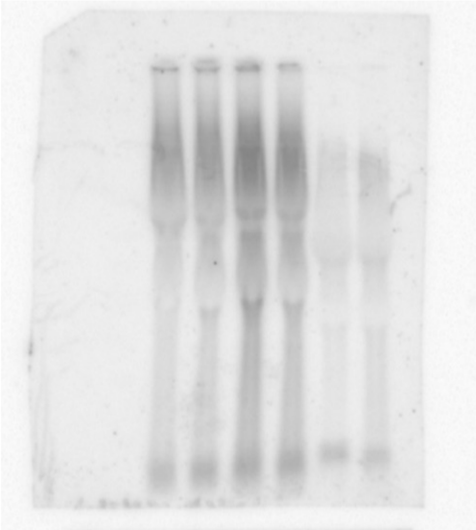

- Probed membrane with GAPDH using the classical approach

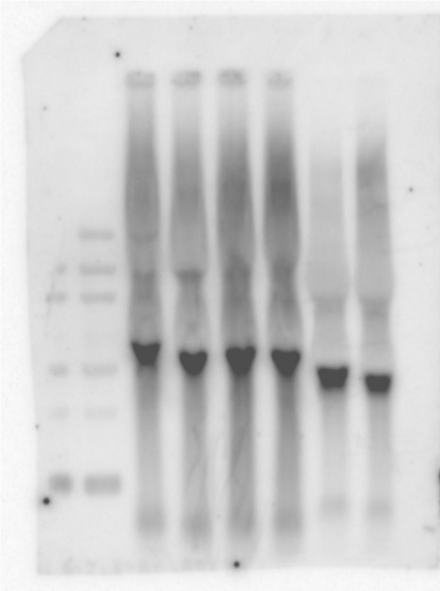

Figure 4E-G

- RNA isolated from ES2 cells

Gel Before Capillary Transfer

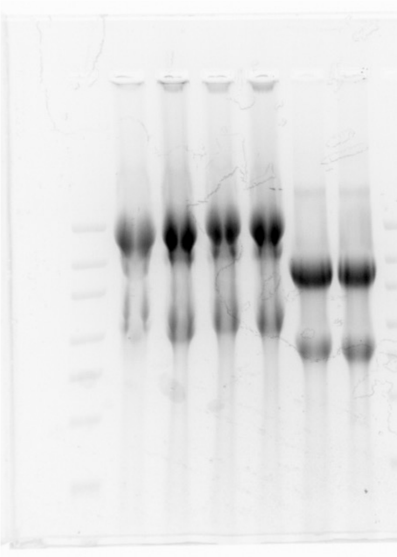

Gel After Capillary Transfer

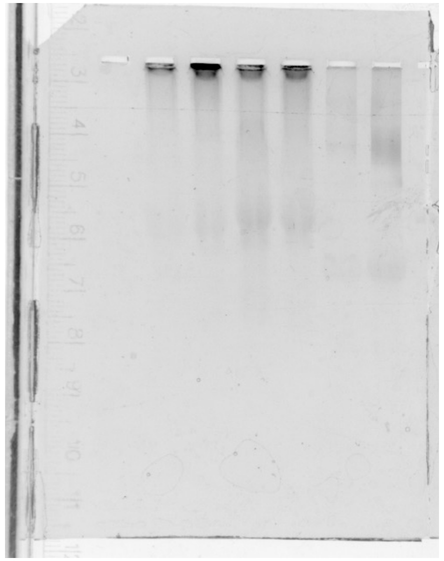

Membrane After Capillary Transfer

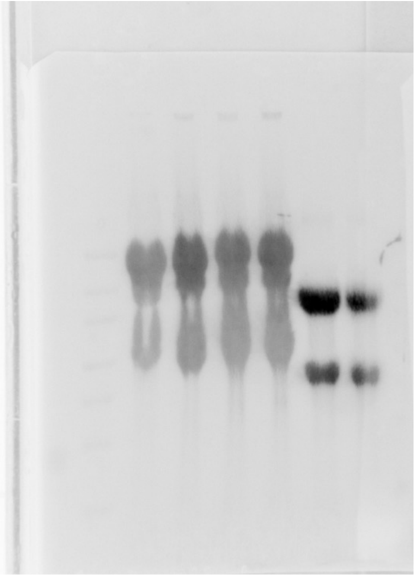

Figure 4H

- Probed membrane with L1-Hs using the alternative approach

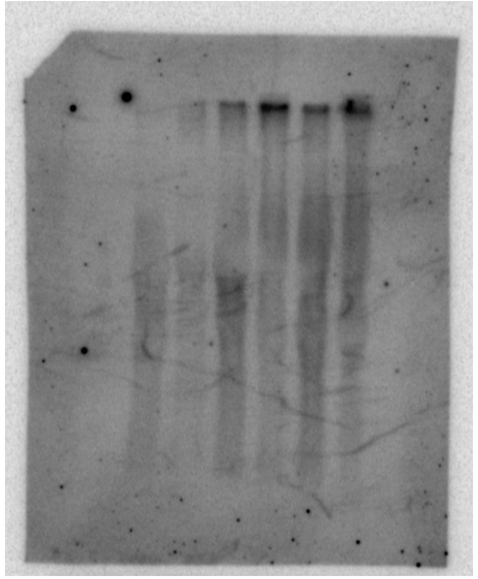

- Probed membrane with GAPDH using the alternative approach

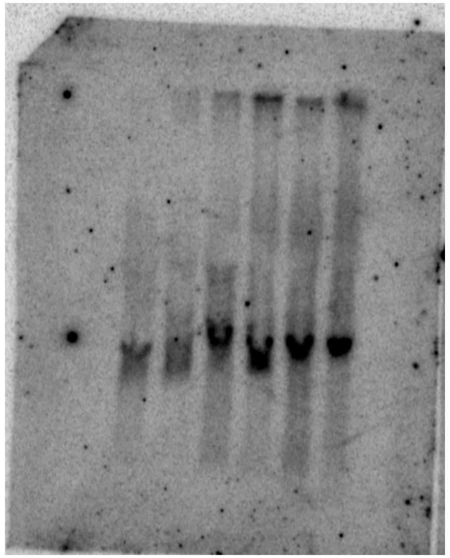

Supplement: bpae036_Supplementary_Data [file bpae036_supplementary_data.zip › Full gels.pdf]
